# Antibacterial and antibiofilm activity of Lactobacillus strains secretome and extraction against *Escherichia coli* isolated from urinary tract infection

**DOI:** 10.1016/j.btre.2022.e00760

**Published:** 2022-08-27

**Authors:** Nayemeh Soltani, Samane Abbasi, Sevda Baghaeifar, Elham Taheri, Mahdieh Farhoudi Sefidan Jadid, Parisa Emami, Kamilia Abolhasani, Firouz Aslanshirzadeh

**Affiliations:** a Department of Microbiology, Urmia Branch, Islamic Azad University, Urmia, Iran; b Department of Biology, Faculty of Sciences, University of Guilan, Rasht, Iran; c Department of Biotechnology, Urmia Branch, Islamic Azad University, Urmia, Iran; d Department of Pharmaceutical Biotechnology, Tabriz University of Medical Sciences, Tabriz, Iran; e Department of Genetics, Tabriz Branch, Islamic Azad University, Branch, Iran; f Department of Genetics, Ahar Branch, Islamic Azad University, Ahar, Iran; g Department of Anesthesia, Tabriz University of Medical Sciences, Tabriz, Iran; h Department of Infectious Disease, Tabriz University of Medical Sciences, Tabriz, Iran

**Keywords:** Anti-bacterial agents, Probiotics, Lactobacillus, Urinary tract infection

## Abstract

The purpose of this study was to antibacterial, and antibiofilm activity of two Lactobacillus strains secretome and extraction against *E. coli* isolated from women with urinary tract infection (UTI). We isolated 100 *E. coli* samples from women with UTI. *Lactobacillus acidophilus* and *Lactobacillus casei* characteristics were evaluated, and their secretome and extraction were prepared. The antibacterial and antibiofilm activity of secretome and extraction of both Lactobacillus strains were evaluated against isolated *E. coli* samples. *L. acidophilus* and *L. casei* were able to tolerate pH 3, bile salts, and pancreatic enzymes. Both probiotics were not resistant to antibiotics and demonstrated an appropriate ability to adhere to the intestinal epithelial cells. Secretome and extraction of *L. acidophilus* and *L. casei* strains showed a good antibacterial and antibiofilm against *E. coli* isolates. Generally, present study suggested that the secretome and extraction of *L. acidophilus* and *L. casei* strains exhibits a good antimicrobial activity.

## Highlights

• We evaluated antibacterial and antibiofilm activity of Lactobacillus strains against *E. coli* isolated from women with UTI.
• *L. acidophilus* and *L. casei* were able to tolerate pH 3, bile salts, and pancreatic enzymes and adhere to intestinal epithelial cells.
• *L. acidophilus* and *L. casei* strains showed a good antibacterial and antibiofilm against *E. coli* isolates with resistant to antibiotics.

## 1 Introduction

Urinary tract infection (UTI) is one of the important health problem in women, which usually is occurred due to *E. coli*. Approximately, 150,000,000 individuals are diagnosed with UTI per year worldwide [1]. UTI is frequently observed with urological problems, which can lead to hypertension of renal failure continued [2]. In fact, a limited serotypes of *E. coli* can cause UTI; these serotypes have been observed with a high tissue invasion, colonization, and adhesion characteristics as compared to other non-pathogenic microorganisms [3, 4].

The major problem in treatment of UTI is related to acquire resistance to common antibiotics through various mechanisms such as changes in efflux pump, outer membrane permeability, target modification, and antibiotic inactivating [5, 6]. Previous studies have reported a high antibiotic resistance (especially to fluoroquinolones and beta-lactam antibiotics) in *E. coli* isolated from patients with UTI [7, 8]. Due to increased antibiotic resistance worldwide and emergence of multi-drug resistant strains, many studies have been conducted to introduce natural compounds as antimicrobial agents [8].

Lactic acid bacteria (LAB) are non-spore forming gram positive and non-respiratory probiotics which produce lactic acid by carbohydrates fermentation. Lactobacillus are an important group of microorganisms that known as probiotic, and recently considered as an antibacterial agent for treatment of human infection [9]. Due to antibacterial effects on pathogenic microorganisms, probiotics are well-known. The antibacterial effects of probiotics are due to production of organic acids (antagonize pathogens), adherence to pathogens, and reduction of bacterial adherents [10, 11]. Moreover, previous studies reported that Lactobacilli bacteria produce several bactericidal compounds [12, 13]. The evidence suggested that production of organic acids and bacteriocin are the major cause of antibacterial activity by Lactobacillus strains [14], [15], [16].

The purpose of this study was to assess antibacterial activity of *Lactobacillus acidophilus* and *Lactobacillus casei* secretome and extractions as probiotic, against *E. coli* isolated from women with UTI.

## 2 Materials and methods

### 2.1 Sample collection

In the present study, 100 *E. coli* isolated from patients with UTI were evaluated, which were identified using the biochemical analyzes. The isolates obtained from women with UTI referred to Asadabadi hospital, Tabriz, Iran. The urine samples of studied patients were cultured in eosin methylene blue (EMB) agar medium. To identification of *E. coli* isolates, the bacterial colonies were evaluated using biochemical analysis, including Voges-Proskauer, methyl red, triple sugar iron (TSI) agar, indole, and citrate. The isolated *E. coli* were cultured in tryptic soy broth (TSB) medium supplemented by glycerol (40%), and then were stored at −20 °C.

### 2.2 Samples isolation and identification

Genomic DNA was extracted using the salting-out method, and amplification of 16sRNA gene was conducted using polymerase chain reaction (PCR) to molecular identification of isolated *E. coli*. The used primers: F: 5`-ACTCTGTTATTAGGGAAGAA-3` and R: 5`-AACGCTTGCCACCTACGTAT-3`. Amplification was conducted in 25 μL total volume: 2.5 μL PCR buffer, 1 μL extracted DNA (50 ng), 1 μL primers (25 pmol), Taq DNA polymerase (1.5 unit), Mgcl2 (1.5 mmol/L), and dNTP (0.1 mmol), in the following condition: initial denaturation: 1 cycle (4 min at 95 °C), denaturation: 35 cycles (1 min at 94 °C), annealing: 35 cycles (1 min at 53 °C), extension: 35 cycles (1 min at 72 °C), and final extension: 1 cycle (10 min at 72 °C). The products of PCR were separated on 2% agarose gel and evaluated by gel document.

### 2.3 Preparation of Lactobacillus strains

Two Lactobacillus strains, include *L. casei* (ATCC 393) and *L. acidophilus* (ATCC 4356) were purchased strains from Persian Type Culture Collection (PTCC). The suspensions of Lactobacillus strains were prepared as follows: lyophilized culture (5 μL) added to 5 mL tryptic soy broth (TSB) and 5 mL de Mann Rogosa and Sharpe broth (MRS), and then standardized using visible-ultraviolet spectrophotometer (600 nm).

### 2.4 Preparation of Lactobacillus secretome and extraction

The Lactobacillus strains was cultured on the de Man, Rogosa and Sharpe (MRS) agar medium. The obtained colonies were inoculated into liquid MRS medium and incubated for 24 h. The bacterial culture was sub-cultured in fresh MRS medium and its absorbance was adjusted on 1 at 600 nm. The obtained bacterial culture was centrifuged and the supernatant was sterilized using a 0.22 μm syringe filter. The different concentrations of supernatant were prepared using MRS broth. Also, the same concentrations of MRS medium were prepared and considered as negative controls.

Moreover, the bacterial plate was resuspended by phosphate buffered saline (PBS) and lysized using ultrasonic bath. The obtained bacterial lysates were sterilized using a 0.22 μm syringe filter. The different concentrations of bacterial extract were prepared using PBS.

### 2.5 Evaluation of acid tolerance of probiotic

The Lactobacillus strains (7–8 log CFU/mL PBS) were inoculated into PBS (pH 3) and PBS (pH 7.2), and incubated for 3 h anaerobically at 37 °C. Next, serial dilutions of each strains were prepared by PBS. Then, 100 µL from bacterial suspension was spread plated on MRS agar and incubated anaerobically for 24 h at 37 °C. The obtained bacterial colonies on MRS agar were enumerated as CFU/mL. The acidic tolerance was evaluated by viability of Lactobacillus strains counts after exposure to normal condition and acidic condition (pH 3). This assay was in triplicate repeat.

### 2.6 Evaluation of bile tolerance of probiotic

Overnight culture of the Lactobacillus strains (adjusted to a 7–8 log CFU/mL) was cultured in MRS broth (10 mL) in presence or absence of oxgall (0.3%), and then incubated anaerobically for 4 h at 37 °C. Next, we prepared serial dilutions tenfold (up to 10^−7^) by PBS. The diluted sample (100 µL) was cultured on MRS agar medium, and then incubated as previous condition. Next, viability of Lactobacillus strains was evaluated by colony counts (CFU/mL). The bile tolerance was evaluated by viability of Lactobacillus strains counts in presence or absent of bile (oxgall).

### 2.7 Evaluation of pancreatic enzyme tolerance of probiotic

Harvested cell pellet of overnight culture of the Lactobacillus strains were resuspended by PBS (7–8 log CFU/mL), and the resuspended cells (1%) was cultured in 10 mL prepared solution (1.9 mg/mL pancreatin and 150 mM NaHCO3 were diluted in PBS, pH 8) and control solution (PBS, pH 7.2), and then incubated anaerobically for 3 h at 37 °C. Next, we prepared serial dilutions tenfold (up to 10^−7^) by PBS. The diluted sample (100 µL) was cultured on MRS agar medium, and then incubated anaerobically for 24 h at 37 °C. The viability of Lactobacillus strains was evaluated by enumeration of colonies (CFU/mL). The pancreatic enzymes tolerance was evaluated by viability of Lactobacillus strains counts in presence or absent of pancreatin (prepared solution).

### 2.8 Evaluation of adherence of probiotic

Human intestinal epithelial cell line (HT-29) was used to probiotic adherence investigation. The cancer cells were cultured using RPMI-1640 medium contains 10% fetal bovine serum (FBS), 1% penicillin-streptomycin (5000 units/mL–5000 mg/mL) antibiotics. Next, the cancer cells (1 × 10^5^/well) were seeded in 6-well plate containing fresh culture medium and incubated at 37 °C in 5% CO_2_. In addition, the overnight culture of Lactobacillus strains (10 mL) were harvested, and then resuspended in sterile PBS at 8 log CFU/mL concentration. The bacterial suspension (100 µL) and fresh culture medium (2 mL) was added to the all wells and then incubated for 1 h. Then, the cells monolayer was washed with PBS, and fixed with methanol (3 mL), and placed at room temperature for 10 min. The cells monolayer was Gram stained and evaluated using a light microscope at 20 random microscopic fields. This assay was in triplicate repeat.

### 2.9 Evaluation of antibiotic susceptibility

The antibiotic susceptibility of Lactobacillus strains was by disc diffusion method. The used antibiotics included: streptomycin (10 μg/ml), ampicillin (10 μg/ml), tetracycline (30 μg/ml), kanamycin (25 μg/ ml), and erythromycin (15 μg/ml). The cultures of Lactobacillus strains (100 μl) was swabbed on surface of nutrient agar medium. Then, the antibiotic discs were placed on the plates. The plates were anaerobically incubated at 37 °C for 24 to 48 h. The diameters of inhibition zones were investigated using calipers and considered as susceptible, S (≥21 mm), resistance, R (≤15 mm), intermediate, I (16–20 mm).

### 2.10 Evaluation of antibacterial activity by microdilution method

Antibacterial activity of the Lactobacillus strains was assessed by a broth microdilution susceptibility test. Briefly, 100 μL of the diluted (1:2 through 1:512) Lactobacillus strains was transferred to a 96-well plates in presence of LB broth medium. The prepared suspension (10^8^ CFU/ml) was then cultured in a 96-well plate, and then incubated for 24 h at 37 °C. Next, the optical density each well (OD) was measured at 620 nm. Finally, samples were serially diluted by PBS and cultured on LB agar medium (in triplicate repeat).

### 2.11 Evaluation of antibacterial activity by disk-diffusion method

Antibacterial activity of the Lactobacillus strains was assessed by disk diffusion method in Mueller Hinton agar medium. Bacterial inoculums were spread plated on Mueller-Hinton agar. Next, empty Whatman discs were impregnated with different concentration of both Lactobacillus strains secretome and extraction and were placed on the plates. The plates were anaerobically incubated at 37 °C for 24 to 48 h. The diameters of inhibition zones were investigated using calipers.

### 2.12 Evaluation of antibiofilm activity by microtiter plate-crystal violet method

Antibiofilm effects of the both Lactobacillus strains were investigated using microtiter plate-crystal violet assay. For this purpose, serially diluted (1:2 through 1:512) strains were cultured in 96-well plates containing LB broth and sucrose. In addition, the *E. coli* strain suspension (10^8^ CFU/ml) was added to 96-well plates and incubated for 24 h at 37 °C. Next, all wells were stained with crystal violet, and de-stained with 95% ethanol. Finally, optical density (OD) of biofilm-related crystal violet was investigated at 570 nm wavelength.

### 2.13 Statistical analysis

In the present study, all experiments were performed in triplicate, and results were presented as mean ± standard deviation (SD). We used SPSS (ver. 21.0) and GraphPad Prism (ver. 6) softwares to analyze of the obtained data. The statistical analysis was performed using Tukey's multiple comparison tests and one-way ANOVA analysis. The *p* < 0.05 was considered as statistically significant.

## 3 Results and discussion

### 3.1 Acid tolerance of probiotic

Both studied Lactobacillus strains showed a high acid tolerance, but the level of tolerance varied among two strains. In present study, *L. acidophilus* showed high acid tolerance (viability loss: 0.16 log units). The acid tolerance of *L. acidophilus* strain was significantly higher than *L. casei* strain (viability loss: 0.30 log unit) (*p* < 0.05). The viability of the Lactobacillus strains at pH 3 and pH 7.2 presented in Table 1. In a similar study, Mourad and Nour-Eddine have indicated that *L. plantarum* isolated from fermented olives showed survival percentages of 55–65%, when exposed to pH 3 for 3 h [17]. Moreover, our results are in agreement with study of Rajoka et al. and Akalu et al., which reported that the isolated Lactobacillus strains from various sources presents above 80% survival rate at pH 3 for several hours [18, 19]. Other previous studies reported that Lactobacillus strains showed a high survival rate (more than 89%) at pH 3 for several hours [20]. However, survival rate of both Lactobacillus strains were significantly decreased at low acidity.

**Table 1:** The acid tolerance of Lactobacillus strains.

Strains	Cell viability (log CFU/mL) ± SD		Viability loss (log units)
	pH 7.2	pH 3.0	
*L. acidophilus*	7.15 ± 0.10	6.99 ± 0.07	0.16 ^a^
*L. casei*	7.03 ± 0.03	6.73 ± 0.11	0.30 ^b^

Standard Deviation (SD); The values with different superscript letters are significantly different (*p* < 0.05).

### 3.2 Bile tolerance of probiotic

Both studied Lactobacillus strains showed a high bile tolerance, but the level of tolerance varied among two strains. The *L. acidophilus* showed the highest tolerance to bile salt (viability loss: 0.12 log units). Moreover, we observed a slight reduction in cell viability of *L. casei* strain (viability loss: 0.44 log units). However, the bile salt tolerance levels of *L. acidophilus* strain were significantly higher than *L. casei* strain (*p* < 0.05). The bile tolerance of the Lactobacillus strains presented in Table 2. Similar to our study, other studies have reported that the isolated Lactobacillus strains are indicate high bile salt tolerance with 88–92% survival rates [21, 22]. In a study by Akalu et al. reported that the isolated Lactobacillus strains are with high tolerance in presence of 0.3% bile salt [18]. In contrast, Boke et al. demonstrated that Lactobacillus strains presents a low levels of bile salts tolerance with decreased survival rates [21]. In addition, Rajoka et al. demonstrated that the Lactobacillus isolates presents a low levels of bile salts tolerance with less than 50% survival rate in presence of bile salts [23].

**Table 2:** The bile tolerance of Lactobacillus strains.

Strains	Cell viability (log CFU/mL) ± SD		Viability loss (log units)
	MRS	MRS + bile salt	
*L. acidophilus*	7.71 ± 0.13	7.59 ± 0.01	0.12 ^a^
*L. casei*	7.93 ± 0.01	7.49 ± 0.08	0.44 ^b^

Standard Deviation (SD); The values with different superscript letters are significantly different (*p* < 0.05).

### 3.3 Pancreatic enzyme tolerance of probiotic

Both studied Lactobacillus strains exhibited an appropriate tolerance to pancreatic enzymes. The *L. acidophilus* showed highest tolerance to the pancreatic enzymes (viability loss: 0.29 log units). The pancreatic enzymes tolerance of *L. acidophilus* strain was higher than *L. casei* strain (viability loss: 0.35 log unit), but this difference was not statistical significant (*p* > 0.05). The pancreatic enzyme tolerance of the Lactobacillus strains presented in Table 3. Pancreatic enzymes in the small intestine are involved in digestion of carbohydrates, fats, and proteins of foods. Tolerate to pancreatic enzymes is another selection criterion to use probiotics [24, 25]. In a similar study by Rönkä et al. reported that the survival rate of L. brevis strain was decreased slightly in presence of pancreatic enzymes [24]. Moreover, Ruiz-Moyano et al. also reported that more than 90% tested Lactobacillus strains survived after 3 h of treating with pancreatic enzymes [26].

**Table 3:** The pancreatic enzyme tolerance of Lactobacillus strains.

Strains	Cell viability (log CFU/mL) ± SD		Viability loss (log units)
	Control	Pancreatic enzymes	
*L. acidophilus*	7.88 ± 0.21	7.59 ± 0.01	0.29 a
*L. casei*	7.71 ± 0.11	7.36 ± 0.12	0.35 a

Standard Deviation (SD); The values with different superscript letters are significantly different (*p* < 0.05).

### 3.4 Adherence assay of probiotic

Both studied Lactobacillus strains were adhered to the HT-29 intestinal epithelial cell line, but adherence ability was different in two Lactobacillus strains. The highest adherence ability was exhibited by *L. casei* (51.8 attached cells per HT-29 cell), and the lowest adherence ability was exhibited by *L. acidophilus* (29.3 attached cells per HT-29 cell). However, this difference was not statistical significant (*p* > 0.05). Ability to attach to intestine epithelial cells and colonize is an important criterion for selection of probiotic isolates which can be established in the intestine [23]. In addition, adherence to intestine epithelial cells is essential probiotics activity, such as antimicrobial activities, cholesterol lowering activity, and immune-modulation. In this study, we used HT-29 cell line (human intestinal cell line) for attachment of two Lactobacillus strains, due to its similar physiological and morphological characteristics to normal human intestine epithelial cells [27]. In a related study by Jacobsen et al. reported variable adhere ability (from strong to low adhesion) by several Lactobacillus strains to HT-29 cell line [28]. Gopal et al. also found that L. acidophilus exhibited strong ability to adhere to the HT-29 human epithelial cell lines [25]. Previous studies demonstrated that the adhesion molecules, exopolysaccharides, on the cell walls were involved in adherence ability of Lactobacillus strains [29, 30]. Moreover, various adhesion factors of Lactobacillus strains are loosely bound to the epithelial cells surface by noncovalent interaction [31].

### 3.5 Antibiotic susceptibility

The antibiotic susceptibility of Lactobacillus strains demonstrated sensitivity to, erythromycin, and tetracycline, ampicillin. However, both Lactobacillus strains showed a resistance to streptomycin and kanamycin. The antibiotic susceptibility of the Lactobacillus strains presented in Table 4. Resistant to antibiotics is a main characteristic of probiotic bacteria. In another study by Tigu et al. reported that isolated Lactobacillus strains from fermented condiments were sensitive to tetracycline, ampicillin, and erythromycin [32]. On the contrary, Sukmarini et al. reported that isolated Lactobacillus strains from Indonesian fermented foods were resistant to erythromycin [30]. In addition, Pan et al. reported that isolated Lactobacillus strains from Chinese fermented foods were resistant to erythromycin, ampicillin, and tetracycline [31].

**Table 4:** The antibiotic susceptibility profile of Lactobacillus strains.

Antibiotics	Dose (μg/ml)	Lactobacillus strains	
		*L. acidophilus*	*L. casei*
Streptomycin	10	R	R
Ampicillin	10	R	R
Tetracycline	30	S	S
Kanamycin	25	R	R
Erythromycin	15	S	S

Susceptible: S (≥21 mm); Intermediate: I (16–20 mm); Resistance: R (≤15 mm).

### 3.6 Antibacterial activity of probiotic

The secretome and extraction of Lactobacillus strains was shown to inhibit the growth of the *E. coli* isolates. According to the obtained results, the extraction of *L. acidophilus* showed a high antibacterial activity. The extraction of *L. casei* presented a high antibacterial activity against the *E. coli* isolates. Moreover, the extraction of both Lactobacillus strains showed a high antibacterial activity than secretome.

The obtained results showed that the largest growth inhibition zone was related to the extraction of *L. acidophilus* (16 mm) and *L. casei* (15 mm) stains. Moreover, the secretome of both Lactobacillus strains showed a larger growth inhibition zone. However, the enrofloxacin created the largest inhibition zone in the *E. coli* isolates (Table 5).

**Table 5:** Inhibition zone diameter of secretome and extraction of Lactobacillus strains on *E. coli* isolates.

Bacterial isolate	*L. acidophilus*		*L. casei*		Enrofloxacin
	Extraction	Secretome	Extraction	Secretome	
*E. coli*	16 mm	9 mm	15 mm	6 mm	23 mm
*E. coli* (PTCC 43,889)	19 mm	12 mm	17 mm	9 mm	26 mm

In a similar study by Bassyouni et al. reported that isolated Lactobacillus strains from fermented condiments presents an antibacterial activity against *E. coli* [33]. Similarly, our study showed a 16 mm inhibition zone diameter in the UTI isolated *E. coli* by extraction of *L. acidophilus*. In agreement to the present study, Tadesse et al. reported that the Lactobacillus isolates can inhibit the *E. coli* strain with 15–17 mm inhibition zone diameters [34]. Tigu et al. have also revealed that Lactobacillus isolates inhibited the growth of *E. coli* with inhibition zones ranging from 10 to 14 mm in diameters [32]. In line with this, Haghshenas et al. have reported that Lactobacillus isolated from Iranian fermented dairy products, Lactobacillus species showed the most efficient antibacterial activity against E*. coli* with inhibition zones of 12.3 mm diameters [22].

### 3.7 Antibiofilm activity of probiotic

**Fig. 1:**
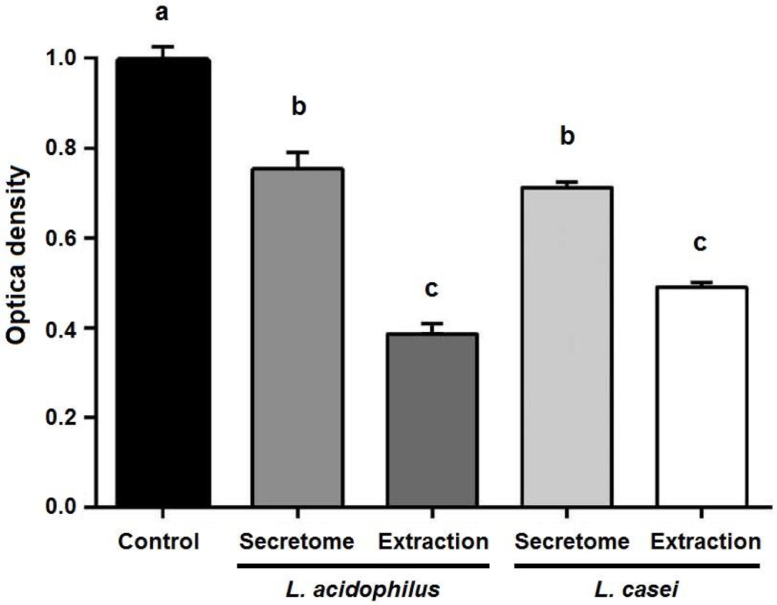
The antibiofilm activity of secretome and extraction of Lactobacillus strains on *E. coli* isolates. The values with different superscript letters are significantly different (*p* < 0.05).

The secretome and extraction of Lactobacillus strains was shown to inhibit biofilm formation of the *E. coli* isolates. The extraction of *L. acidophilus* and *L. casei* strains showed a high antibiofilm activity than their secretome (Fig. 1). This finding indicated that, the *L. acidophilus* and *L. casei* probiotics used in the present study had ability to inhibit biofilm formation by *E. coli* isolates. In parallel with our findings, Rao et al. have declared that cell free supernatant of Lactobacillus strains showed good antibiofilm activity [35]. Moreover, Khiralla et al. reported that three Lactobacillus strains isolated from traditional products are strongly recommended as biocontrol agents by inhibition of pathogens ability to form biofilm [36].

## 4 Conclusion

In conclusion, we suggested that the Lactobacillus strains in the present study displayed potential probiotic properties. These strains had significant antimicrobial effect against *E. coli* isolated from patients with UTI. Moreover, we showed the antibiofilm effect of Lactobacillus strains against *E. coli* isolates. The effects of *L. acidophilus* and *L. casei* probiotics are not limited only to promote of human healthy, it also provides antibacterial effect against pathogenic bacteria. The results of this study indicated that *L. acidophilus* and *L. casei* probiotics directly interact with cancer cells and indirectly inhibit growth of E. coli isolate by release various bacitracin and metabolites. However, further studies are needed to investigate probiotic characteristics of various Lactobacillus strains.

## Declaration of Competing Interest

The authors declare that they have no competing interests.
